# Enzymatically dissociated muscle fibers display rapid dedifferentiation and impaired mitochondrial calcium control

**DOI:** 10.1016/j.isci.2022.105654

**Published:** 2022-11-22

**Authors:** Charlotte Gineste, Sonia Youhanna, Sabine U. Vorrink, Sara Henriksson, Andrés Hernández, Arthur J. Cheng, Thomas Chaillou, Andreas Buttgereit, Dominik Schneidereit, Oliver Friedrich, Kjell Hultenby, Joseph D. Bruton, Niklas Ivarsson, Linda Sandblad, Volker M. Lauschke, Håkan Westerblad

**Affiliations:** 1 Department of Physiology and Pharmacology, Karolinska Institutet, 171 77 Stockholm, Sweden; 2 Umeå Core Facility for Electron Microscopy, Department of Chemistry, Umeå University, 901 87 Umeå, Sweden; 3 Institute of Medical Biotechnology, Department of Chemical and Biological Engineering, Friedrich-Alexander University of Erlangen-Nürnberg, 91052 Erlangen, Germany; 4 Department of Laboratory Medicine, Karolinska Institutet, Karolinska University Hospital Huddinge, 141 86 Huddinge, Sweden; 5 Dr. Margarete Fischer-Bosch-Institute of Clinical Pharmacology, Stuttgart, Germany; 6 University of Tübingen, Tübingen, Germany

**Keywords:** Cellular physiology, Cell biology, Functional aspects of cell biology, Developmental biology

## Abstract

Cells rapidly lose their physiological phenotype upon disruption of their extracellular matrix (ECM)-intracellular cytoskeleton interactions. By comparing adult mouse skeletal muscle fibers, isolated either by mechanical dissection or by collagenase-induced ECM digestion, we investigated acute effects of ECM disruption on cellular and mitochondrial morphology, transcriptomic signatures, and Ca^2+^ handling. RNA-sequencing showed striking differences in gene expression patterns between the two isolation methods with enzymatically dissociated fibers resembling myopathic phenotypes. Mitochondrial appearance was grossly similar in the two groups, but 3D electron microscopy revealed shorter and less branched mitochondria following enzymatic dissociation. Repeated contractions resulted in a prolonged mitochondrial Ca^2+^ accumulation in enzymatically dissociated fibers, which was partially prevented by cyclophilin inhibitors. Of importance, muscle fibers of mice with severe mitochondrial myopathy show pathognomonic mitochondrial Ca^2+^ accumulation during repeated contractions and this accumulation was concealed with enzymatic dissociation, making this an ambiguous method in studies of native intracellular Ca^2+^ fluxes.

## Highlights

• Collagenase treatment disrupts the complex cytoarchitecture of muscle fibers
• Muscle fibers rapidly dedifferentiate on collagenase-mediated cell isolation
• Dedifferentiation is paralleled by aberrant mitochondrial Ca^2+^ accumulation
• Cyclophilin inhibitors limit the aberrant mitochondrial Ca^2+^ accumulation

## Graphical abstract

**Figure:**
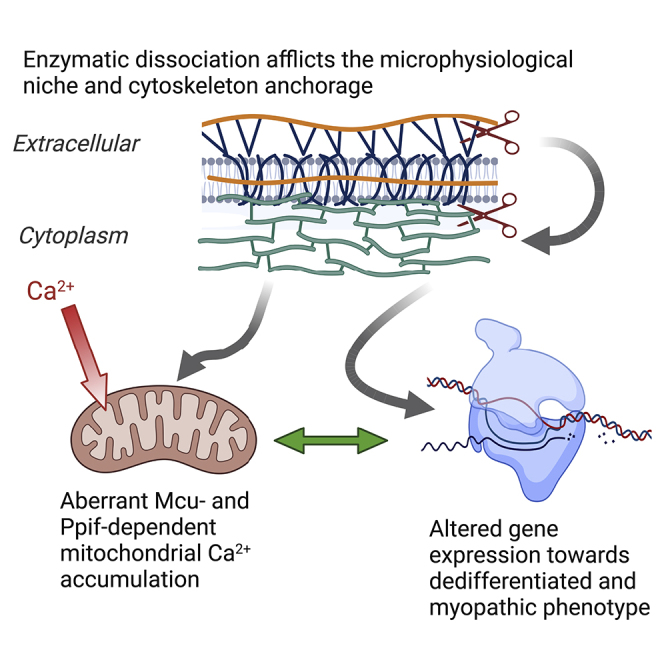

## Introduction

The intracellular secondary messenger Ca^2+^ is of critical importance for a plethora of cellular functions. Within the cell, Ca^2+^ levels are compartmentalized and can differ by orders of magnitude between the cytosol and organelles, such as the sarcoplasmic reticulum (SR). In this context, mitochondrial Ca^2+^ levels have received increasing attention in recent years for their important roles in energy homeostasis, oxidative stress, and apoptosis.^1^^,^^2^^,^^3^ A limited and transient increase in free mitochondrial matrix [Ca^2+^] ([Ca^2+^]_mit_) can stimulate mitochondrial respiration and hence play an integral role in the regulation of cellular metabolism, whereas prolonged and excessive uptake can activate apoptotic and necrotic cell signaling pathways.^4^^,^^5^^,^^6^ Tight control of [Ca^2+^]_mit_ seems particularly important in skeletal and cardiac muscle cells where cytosolic free [Ca^2+^] ([Ca^2+^]_cyt_) can reach μM concentrations, and even higher in the vicinity of SR Ca^2+^ release sites.^7^ Accordingly, an increase in [Ca^2+^]_mit_ in response to increased [Ca^2+^]_cyt_ has been observed during *in vivo* contractions of mouse skeletal muscle, and [Ca^2+^]_mit_ rapidly returned to the basal level after the end of contraction.^8^

In general terms, [Ca^2+^]_mit_ is set by the balance between mitochondrial Ca^2+^ entry, buffering capacity, and extrusion. The mitochondrial Ca^2+^ uniporter (MCU) was recently identified as the pore-forming unit of a mitochondrial Ca^2+^ uptake channel,^9^^,^^10^ whereas mitochondrial Ca^2+^ extrusion is mediated by the Na^+^/Ca^2+^ antiporter in skeletal muscle (Nclx).^11^

Mitochondrial Ca^2+^ entry through MCU is fine-tuned by various regulators.^2^^,^^12^ For instance, the mitochondrial Ca^2+^ uptake protein 1 (Micu1) has been shown to act as gatekeeper for MCU-mediated mitochondrial Ca^2+^-uptake under basal conditions and has a critical role in cellular differentiation, maturation, oxidative metabolism, and damage repair,^13^^,^^14^^,^^15^ as evidenced by loss-of-function mutations of *MICU1* can cause human proximal myopathy.^16^ Intriguingly, muscle function is well maintained in mice lacking Mcu and their [Ca^2+^]_mit_ is reduced but still measurable, which indicates that additional slower, yet undefined uptake mechanisms exist.^17^^,^^18^

Importantly, bidirectional mechanical and biochemical signaling between the extracellular matrix (ECM) and the intracellular cytoskeleton plays a key role in the control of cell structure, including the morphology and function of mitochondria.^19^^,^^20^^,^^21^ In skeletal muscle fibers, mitochondria are tethered to the SR at sites of Ca^2+^ release,^22^ and this spatial organization is required for appropriate Ca^2+^ handling.^23^ Disturbance of the spatial interaction between mitochondria and the SR in muscle cells results in altered Ca^2+^ exchange between the two organelles, which in turn can entail functional impairments in both organelles.^24^ For instance, mutations in desmin, the main protein of the cytoskeleton intermediate filament component, cause a class of myopathies,^25^ and a larger elevation of basal [Ca^2+^]_mit_ after repeated contractions has been observed in skeletal muscle fibers of a transgenic mouse model of desmin myopathy.^26^ Moreover, disruption of crosstalk between the ECM and cytoskeleton because of mutations in ECM components give rise to several muscular diseases,^27^^,^^28^ including collagen VI mutations resulting in Bethlem myopathy, Ullrich congenital muscular dystrophy, and congenital myosclerosis where mitochondrial Ca^2+^ overload is regarded as a key pathogenic factor.^29^ Muscle defects in collagen VI-deficient mice as well as in patients with collagen VI mutation-dependent myopathies are mitigated by pharmacological inhibition of the mitochondrial matrix protein peptidyl-prolyl *cis*-trans isomerase F (mouse Ppif: UniProtKB - Q99KR7; human PPIF: UniProtKB - P30405), a treatment considered to inhibit the Ca^2+^-dependent opening of the mitochondrial permeability transition pore (mPTP).^30^^,^^31^^,^^32^^,^^33^^,^^34^^,^^35^ Note that Ppif is also called cyclophilin D, whereas *PPID* encodes the peptidylprolyl isomerase D, which is a different protein involved in protein folding.

In mammals, each muscle fiber is under the direct control of a single branch of an α-motoneuron and hence its cellular activation occurs independently of the activity of neighboring muscle fibers. Thus, studies on isolated single muscle fibers will reflect the function of muscle fibers *in vivo* and are therefore highly valuable in mechanistic studies of, for instance, the intracellular signaling associated with muscle activation and contraction. Of importance, single muscle fibers are often isolated via collagenase treatment,^23^^,^^36^^,^^37^^,^^38^^,^^39^^,^^40^ which inevitably disrupts the extracellular niche and ECM-cell interactions. Here, we compare collagenase-dissociated mouse muscle fibers with fibers obtained by high-precision mechanical dissection,^41^ and demonstrate that an intact microenvironment is required for maintenance of cellular structure and properly controlled mitochondrial Ca^2+^ handling. Specifically, by integrating data obtained with second harmonic generation (SHG) and immune-fluorescence imaging, electron microscopy, and RNA-sequencing, we show that enzymatic fiber dissociation, but not mechanical microdissection, causes altered cellular organization and the deterioration of molecular signatures characteristic of mature muscle fibers. Furthermore, repeated tetanic contractions resulted in a marked increase of basal [Ca^2+^]_mit_ (i.e., measured at rest and not during an ongoing contraction) in enzymatically dissociated but not in mechanically dissected muscle fibers.

A pronounced contraction-induced increase in basal [Ca^2+^]_mit_ similar to that in enzymatically dissociated fibers has previously been observed in mechanically dissected muscle fibers from a mouse model of severe mitochondrial myopathy, the fast-twitch skeletal muscle fiber-specific mitochondrial transcription factor A knock-out (*Tfam* KO) mouse, and this increase was implied to have a central role in the disease progress.^42^^,^^43^ In the second part of the present study we therefore asked whether an aberrant contraction-induced increase in basal [Ca^2+^]_mit_ in mitochondrial myopathy muscle fibers can be detected in enzymatically dissociated fibers, where basal [Ca^2+^]_mit_ shows a marked increase already in muscle fibers of healthy mice.

## Results

### Intact links between ECM and cytoskeleton are necessary to maintain the structural integrity of skeletal muscle fibers

**Figure 1:**
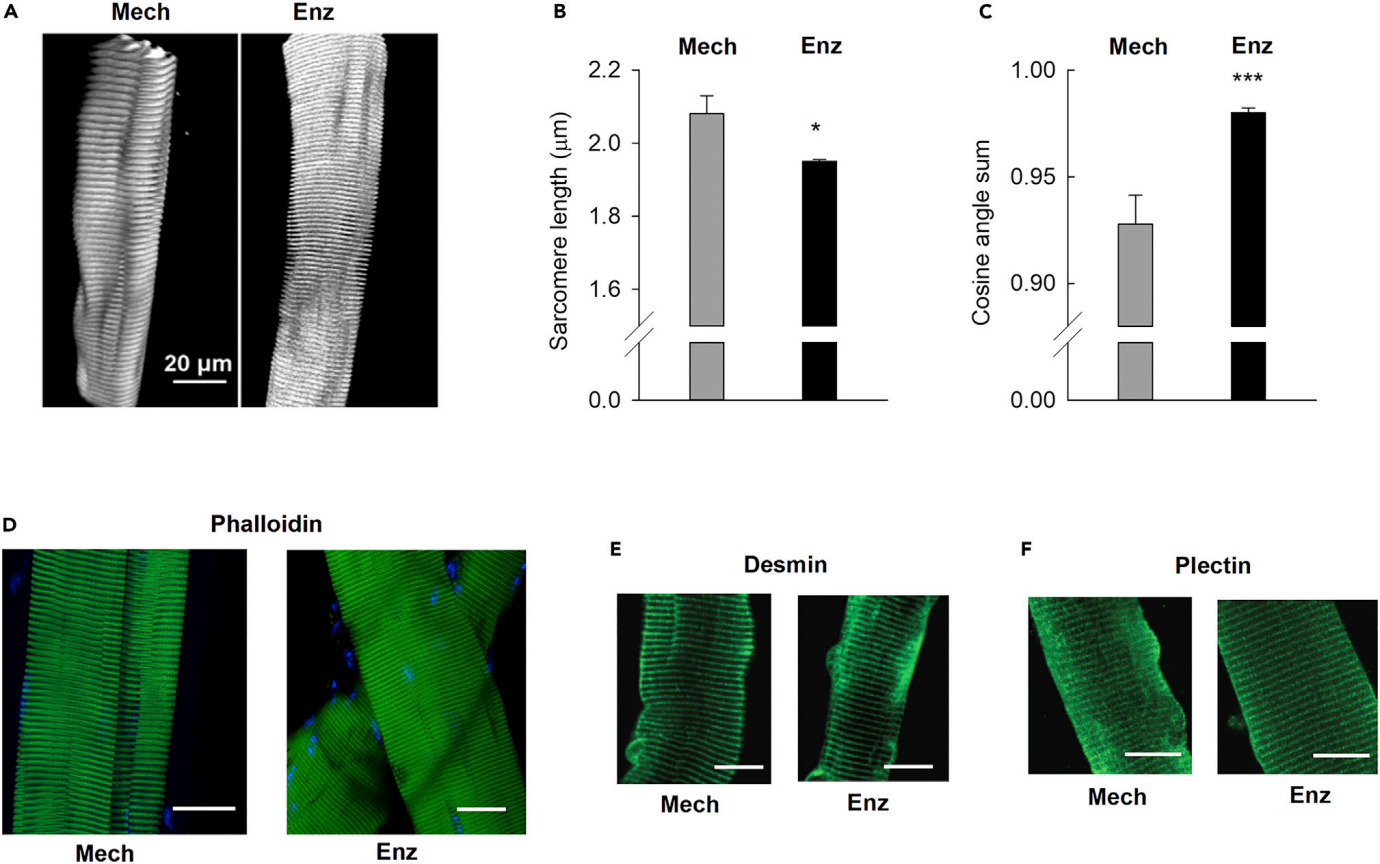
Disruption of the extracellular matrix results in altered cell shape (A) Representative SHG images from a mechanically dissected (Mech) and an enzymatically dissociated (Enz; using collagenase) mouse FDB fiber. Average data of (B) sarcomere length and (C) cosine angle sum (CAS) in mechanically dissected (n = 5) and enzymatically dissociated (n = 13) fibers. CAS of 1 represents a perfectly linear parallel pattern, whereas CAS = 0 reflects perpendicularly oriented structures. Gray bars = mechanically dissected fibers; black bars = enzymatically dissociated fibers. ∗p < 0.05 and ∗∗∗p < 0.001 versus mechanically dissected fibers with unpaired t-test. Data are presented as mean ± SEM. Representative images of phalloidin-labeled actin (D) and immunofluorescence staining of desmin (E) and plectin (F) in mechanically dissected and enzymatically dissociated fibers. Scale bars, 20 μm. Experiments were performed 4 h after muscle fiber isolation.

To examine the effects of the extracellular microenvironment on cellular phenotype, we compared mechanically dissected mouse flexor digitorum brevis (FDB) fibers with fibers isolated by conventional enzymatic dissociation using collagenase. Mechanical microdissection permits the isolation of muscle fibers with tendons attached and intact sarcolemma,^41^ including the adjacent ECM scaffold with preserved focal adhesion complexes. In contrast, physiological ECM contacts are afflicted by collagenase treatment. We first examined whether the different isolation methods translated into differences in cell morphology. To this end, we combined multi-photon-based SHG imaging and a quantitative morphometry technique to assess possible alterations in the myofibrillar architecture induced by the collagenase treatment.^44^ General SHG imaging showed collagen structures attached to mechanically dissected fibers (Video S1), whereas no extracellular collagen was detected on enzymatically dissociated fibers (Video S2). For further analyses, we filtered out the collagen-1 signal and specifically focused on myosin. SHG imaging of single mechanically dissected FDB fibers then revealed a complex myofibrillar architecture with longitudinal ridges and valleys. Enzymatically dissociated fibers, on the other hand, were more homogeneously delineated with almost circular cross-sections and shorter, more evenly extended sarcomeres (Figures 1A and 1B; Videos S3 and S4).

Video S1. SHG 3D imaging of a representative mechanically dissected muscle fiber showing attached collagen structures, related to SHG imaging experiments and Figure 1

Video S2. SHG 3D imaging of a representative enzymatically dissociated muscle fiber showing that extracellular collagen structures are lost with the isolation procedure, related to SHG imaging experiments and Figure 1

Video S3. Myosin-focused 3D imaging of a representative mechanically dissected muscle fiber, related to SHG imaging experiments and Figure 1

Video S4. Myosin-focused 3D imaging of a representative enzymatically dissociated muscle fiber, related to SHG imaging experiments and Figure 1

To further characterize the structural difference between mechanically dissected and enzymatically dissociated fibers, we quantified the cosine angle sum (CAS) in SHG images. CAS represents the summed-up contributions of all projections from local myofibrillar directionality against the main fiber axis and is used as a measure of a fiber’s myofibrillar angular alignment.^44^^,^^45^^,^^46^ Notably, CAS was significantly higher (reflecting a more linear and parallel pattern) for enzymatically dissociated than for mechanically dissected fibers, further corroborating the observation of homogeneously striated patterns and parallel myofibrillar alignment in dissociated fibers (Figure 1C).

We used histochemistry to further characterize the cellular structure and identify possible differences between mechanically dissected and enzymatically dissociated fibers. In alignment with the results from SHG imaging, phalloidin labeling of actin showed distinct longitudinal streaks, reflecting longitudinal ridges and valleys, whereas enzymatically dissociated fibers appeared more homogeneously delineated (Figure 1D). Antibody staining of the cytoskeletal proteins desmin and plectin showed clear cross-striations in both mechanically dissected and enzymatically dissociated fibers (Figures 1E and 1F). In agreement with the results of SHG imaging, sarcomere lengths were longer in enzymatically dissociated (1.92 ± 0.02 μm, n = 57) than in mechanically dissected fibers (1.73 ± 0.03 μm, n = 43; p < 0.001).

### 3D electron microscopy revealed an enzymatic dissociation-induced reduction in volume of individual mitochondria

**Figure 2:**
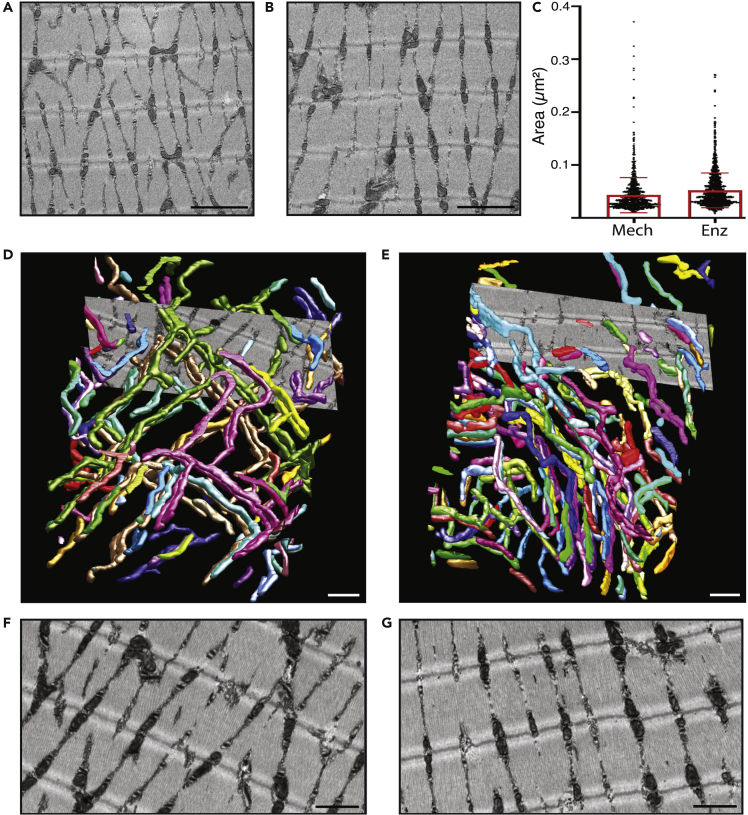
Disruption of the extracellular matrix causes subtle changes in mitochondrial morphology as detected with 3D electron microscopy Representative TEM images of a mechanically dissected (A) and a collagenase dissociated (B) FDB muscle fiber. Scale bar in A and B is 2 μm. (C) Graphical presentation of individual and mean (±SD) mitochondrial areas measured from TEM images acquired from mechanically dissected fibers (left; n = 1154 mitochondria in 20 images of distinct areas of 6 fibers) and enzymatically dissociated fibers (right, n = 1390 mitochondria in 20 images of distinct areas of 10 fibers). 3D model of the mitochondria in a volume of 365 μm^3^ muscle tissue obtained with FIB-SEM in a dissected fiber (D) and collagenase-treated fiber (E); rotating 3D models are presented in Videos S5 and S6. Individual mitochondria are distinguished by different colors. Representative images from slices used in the FIB-SEM-based modeling acquired in the dissected (F) and the collagenase-treated (G) fiber, respectively. Fibers were fixed 4 h after being isolated. Scale bar in (D–G) is 1 μm.

Next, we evaluated whether the collagenase-induced disruption of the extracellular microenvironment would propagate into alterations in mitochondrial spatial organization and ultrastructure. To this end, we analyzed fibers of both mechanically dissected and enzymatically dissociated fibers using transmission electron microscopy (TEM), and 3D mitochondrial models were constructed with focused ion beam scanning electron microscopy (FIB-SEM).^47^^,^^48^ TEM analysis of ultrathin sections showed no significant difference in mitochondrial cross-sectional areas between the two groups (Figures 2A–2C). 3D mitochondrial models of similar fiber volumes (∼365 μm^3^) were constructed from a mechanically dissected and an enzymatically dissociated fiber by sequential FIB milling of 30 nm slices, (Figures 2D–2G). The 3D images look grossly similar in the two fibers with mitochondria preferentially oriented perpendicular to the long axis of the fiber and localized adjacent to the z-discs (Videos S5 and S6), which agrees with the pattern previously observed in glycolytic muscle fibers.^49^ The total mitochondrial volume was similar in the two models (20.2 versus 17.1 μm^3^, corresponding to 5.5% versus 4.6% of the total fiber volume). On the other hand, the number of mitochondria was clearly lower (86 versus 140) and their average volume larger in the mechanically dissected (0.235 μm^3^) than in the enzymatically dissociated (0.122 μm^3^) fiber.

Video S5. FIB-SEM-generated 3D imaging of the mitochondrial network in a mechanically dissected muscle fiber, related to Figure 2

Video S6. FIB-SEM-generated 3D imaging of the mitochondrial network in an enzymatically dissociated muscle fiber, related to Figure 2

### Disruption of the cellular microenvironment causes a rapid deterioration of transcriptomic signatures

**Figure 3:**
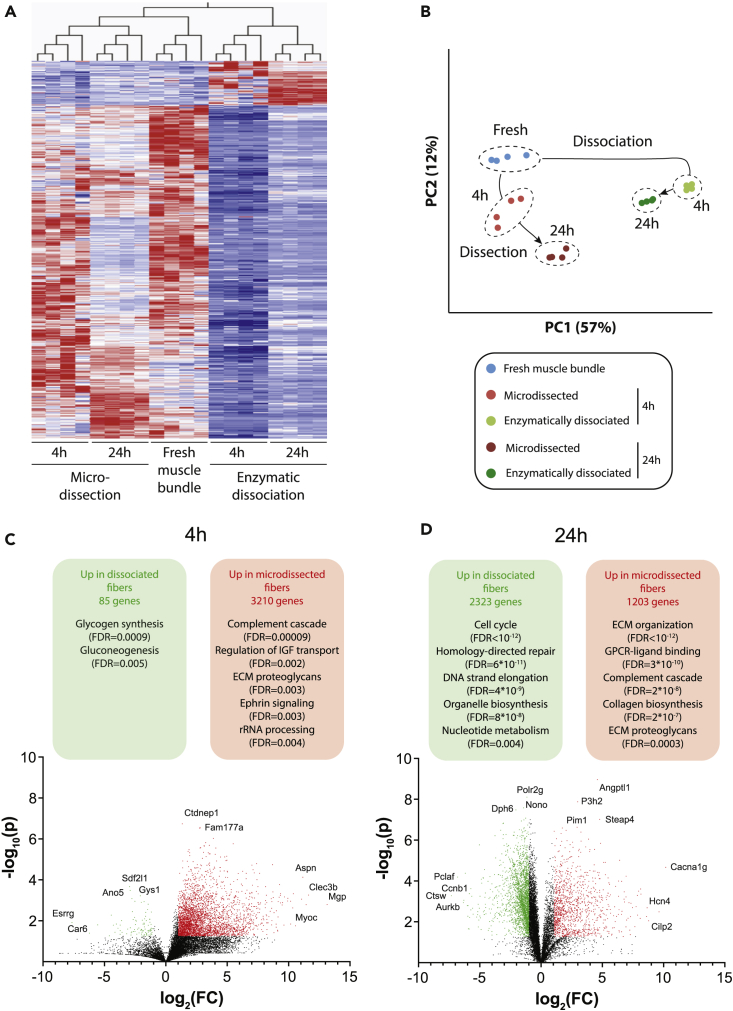
RNA-Sequencing reveals major transcriptomic signature changes in response to muscle fiber isolation (A) Mean-centered, sigma-normalized heatmap visualization of differentially expressed genes (F-test; FDR = 5%; Benjamini-Hochberg correction). Note that microdissected fibers more closely resemble the gene expression patterns of freshly isolated muscle bundles than enzymatically dissociated fibers. (B) The corresponding principal component analysis (PCA) reveals that enzymatic dissociation and microdissection result in orthogonal gene expression alterations. (C and D) Volcano plots visualizing differentially expressed genes between microdissected and enzymatically dissociated fibers 4h (C) and 24h (D) after isolation. Red and green dots indicate genes that are significantly (fold-change [FC]>2 and p < 0.05 in heteroscedastic two-tailed T-test) upregulated in microdissected and enzymatically dissociated fibers, respectively. Significantly enriched pathways corresponding to the differentially expressed genes are indicated in the respective colored boxes.

We used RNA-sequencing to compare the transcriptomic signatures of freshly isolated muscle fiber bundles to those of fibers isolated either by mechanical dissection or by enzymatic dissociation. Notably, we observed striking differences in gene expression patterns depending on the isolation method (Figure 3A). Principal component analysis revealed that overall changes in expression were more rapid and extensive in dissociated fibers (Figure 3B). Compared to freshly isolated muscle fiber bundles, 514 genes were differentially expressed in mechanically dissected fibers, whereas more than twice as many (1156 genes) were altered upon enzymatic dissociation (FDR = 0.01). Systematic comparison of expression differences between isolation methods revealed that after 4h, 3,210 genes showed significantly higher levels in mechanically dissected than in enzymatically dissociated fibers and these were enriched in complement signaling, ECM proteoglycans, ephrin signaling and rRNA processing (Figure 3C). In contrast, only 85 genes showed higher expression in enzymatically dissociated than in mechanically dissected fibers, specifically those involved in glycogen synthesis and gluconeogenesis. Conversely, 24h after isolation, mechanically dissected fibers featured 1,203 significantly upregulated genes mostly related to ECM biogenesis and organization, whereas enzymatically dissociated fibers show upregulation of 2,323 genes significantly enriched in cell cycle, DNA replication and repair (Figure 3D).

**Figure 4:**
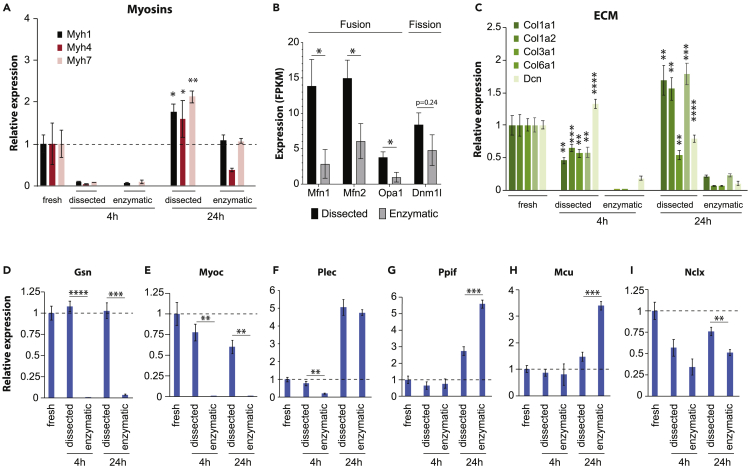
Enzymatic dissociation alters the expression of myosins, extracellular matrix components, the mitochondrial fusion machinery and mitochondrial Ca^2+^ regulators (A) Expression of functionally relevant myosins are significantly downregulated in enzymatically dissociated versus mechanically dissected fibers. (B) The mitofusins Mfn1 and Mfn2, as well as Opa1 are significantly downregulated in dissociated fibers 4h after isolation, whereas expression of the key mitochondrial fission regulator Dnm1l is not significantly altered. (C) Extracellular matrix (ECM) components are significantly downregulated in dissociated fibers. The actin regulators Gsn (D) and Myoc (E), as well as the intermediate filament-associated protein Plec (F) are among the genes whose expression is almost completely lost 4 h after enzymatic dissociation. The gene expression levels of mitochondrial proteins involved in Ca^2+^ uptake, Ppif (G) and Mcu (H), are increased in dissociated fibers, whereas levels of Nclx involved in mitochondrial Ca^2+^ efflux are significantly decreased (I). ∗, ∗∗, ∗∗∗ and ∗∗∗∗ indicate p < 0.05, p < 0.01, p < 0.001 and p < 0.0001 between dissected and dissociated fibers at the same time point. Data are presented as mean ± SEM; n = 4.

The expression of genes encoding for myosin heavy chain IIX (Myh1), IIB (Myh4), and I (Myh7) was substantially reduced after 4h irrespective of isolation method; after 24h, on the other hand, expression had recovered and was significantly higher in mechanically dissected than in enzymatically dissociated fibers (Figure 4A), whereas the gene encoding for myosin heavy chain IIA (Myh2) was not differentially expressed between the two groups (Figure S1A). In enzymatically dissociated fibers, we furthermore observed a rapid and significant downregulation of genes involved in mitochondrial fusion (Mfn1, Mfn2 and Opa1), whereas no difference was observed for the fission component Dnm1l (also referred to as DRP; Figure 4B). These findings are consistent with our observations that enzymatically dissociated muscle fibers featured significantly more but smaller mitochondria and suggest that these differences are likely because of reduced mitochondrial fusion rather than increased fission.

The gene expression of critical ECM components, including the abundant muscle collagens Col1a1, Col1a2, Col3a1, and Col6a1, as well as the proteoglycan decorin (Dcn), were almost entirely lost in enzymatically dissociated fibers at both time points, whereas expression remained relatively stable in mechanically dissected fibers (Figure 4C). Similar effects were observed for the gene expression of gelsolin (Gsn), a calcium-regulated protein involved in the assembly of actin filaments, and myocilin (Myoc), a regulator of cytoskeletal function (Figures 4D and 4E). Expression of the cytoskeletal scaffolding protein plectin (Plec) was also significantly downregulated in enzymatically dissociated fibers after 4h, whereas the expression was increased in both groups at 24 h (Figure 4F).

Of interest, at 24h the expression of *Ppif* and *Mcu* were higher with enzymatic dissociation than with mechanical dissection (Figures 4G and 4H), whereas the expression of *Nclx* was lower in enzymatically dissociated fibers (Figure 4I), hence all three differences acting toward increased mitochondrial Ca^2+^ accumulation in enzymatically dissociated fibers. The expression of Mcu regulators showed no consistent pattern (Figures S1B–S1F). Taken together, these findings confirm that enzymatic dissociation of muscle fibers results in a rapid deterioration of the mature muscle phenotype, approaching signatures that resemble pathological phenotypes with decreased expression of genes encoding for critical ECM elements and ECM-cytoskeletal signal transducers, as well as an activation of regenerative pathways.

### Collagenase treatment results in aberrant mitochondrial Ca^2+^ accumulation

**Figure 5:**
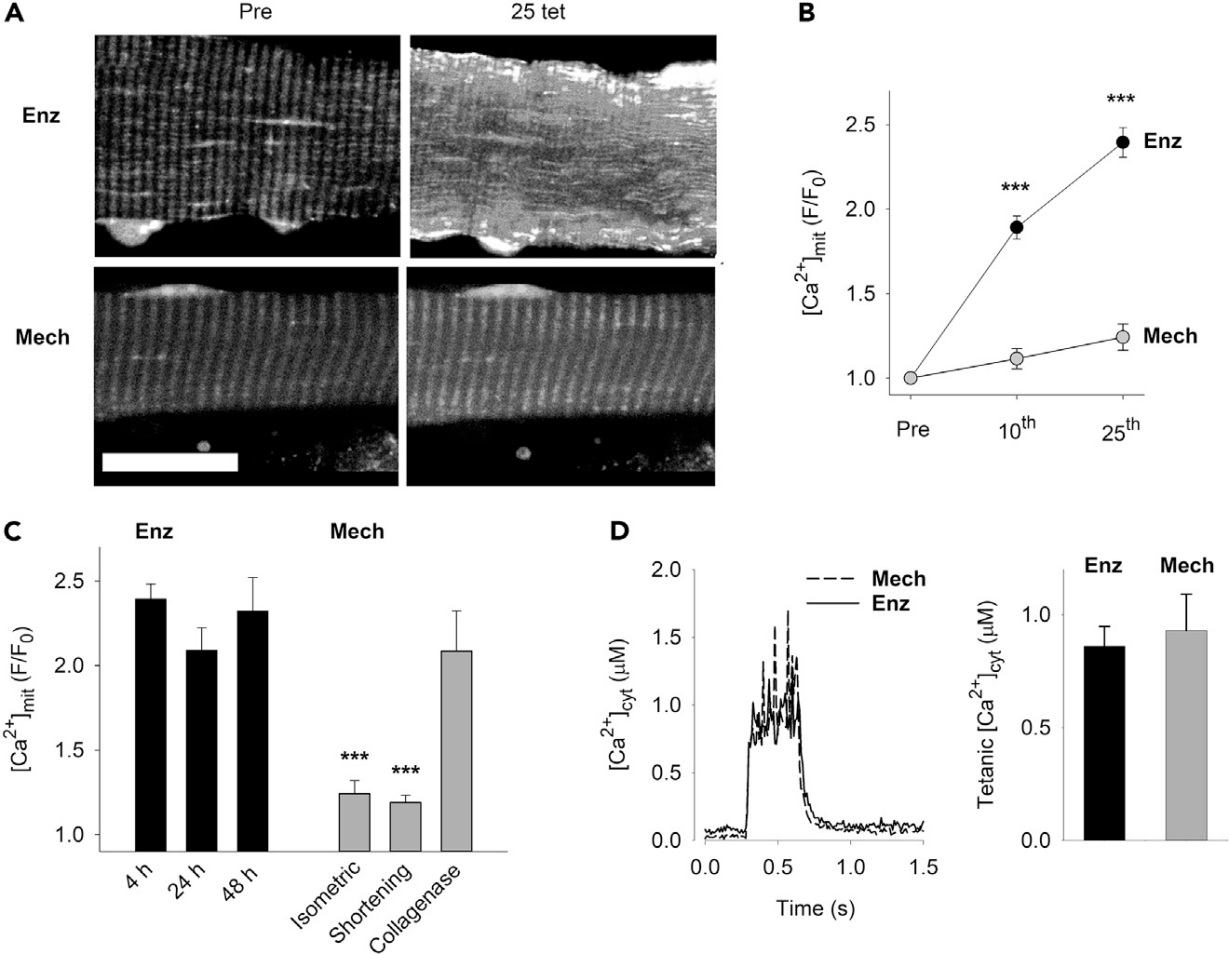
Enzymatic dissociation of muscle fibers results in impaired mitochondrial Ca^2+^ control (A) Representative rhod-2 fluorescence images of enzymatically dissociated (Enz) and mechanically dissected (Mech) mouse FDB fibers before and after 25 brief tetanic contractions. Scale bar, 20 μm. (B) Mean data of rhod-2 fluorescence ∼5 s after (F) relative to before (F_0_) 10 and 25 repeated tetani in Enz (black circles, n = 15) and Mech (gray circles, n = 9) fibers measured 4 h after isolation. ∗∗∗p < 0.001 Enz versus Mech with two-way RM ANOVA. (C) Mean data of rhod-2 fluorescence ∼5 s after (F) relative to before (F_0_) 25 repeated tetani in Enz fibers stored for 4–48 h after isolation (black bars) and in Mech fibers studied 4 h after dissection either when contracting isometrically, shortening freely, or after collagenase treatment (gray bars). ∗∗∗p < 0.001 versus 4 h dissociated fibers with one-way ANOVA(n ≥ 5). (D) Average [Ca^2+^]_cyt_ records (left) and mean tetanic [Ca^2+^]_cyt_ (right) during tetanic stimulation (70 Hz, 350 ms duration) of enzymatically dissociated (solid line and black bar) and mechanically dissected (dashed line and gray bar) FDB fibers (n = 6 in both groups). Data are presented as mean ± SEM.

Next, we investigated whether collagenase-induced disturbance of the ECM would impact mitochondrial Ca^2+^ handling. [Ca^2+^]_mit_ has been shown to rapidly increase during brief contractions of mouse skeletal muscle electrically stimulated *in vivo.*^8^ This increase was transient and [Ca^2+^]_mit_ rapidly returned toward the resting level when stimulation was stopped. Nevertheless, in the majority (5 out of 7) muscle fibers studied, [Ca^2+^]_mit_ did not have time to fully return to basal level between contractions when 500 ms tetani were evoked every ∼2s.^8^ Thus, mitochondria potentially accumulate Ca^2+^ during repeated contractions resulting in a prolonged elevation of basal [Ca^2+^]_mit_ (i.e. measured at rest and not during an ongoing contraction), and we here studied this aspect of mitochondrial Ca^2+^ handling by stimulating isolated FDB muscle fibers at 70 Hz for 350 ms every 2 s. A total of 25 tetanic contractions were evoked and changes in basal [Ca^2+^]_mit_ were assessed from confocal images of the fluorescence of the [Ca^2+^]_mit_ indicator rhod-2 obtained before and ∼5 s after the 10^th^ and last contractions. In mechanically dissected fibers, basal [Ca^2+^]_mit_ did not show any marked increase during the 25 tetanic contractions, which agrees with previous results from FDB fibers of wildtype mice and implies intact mitochondrial Ca^2+^ control^42^ (Figures 5A and 5B). Intriguingly and in sharp contrast to mechanically dissected fibers, the repeated contractions caused a marked increase of basal [Ca^2+^]_mit_ in enzymatically dissociated fibers with rhod-2 fluorescence being increased by ∼90% after 10 tetani and by ∼140% after 25 tetani. The increase in basal [Ca^2+^]_mit_ after 25 tetanic contractions was similar in enzymatically dissociated fibers 4, 24, and 48 h after fiber isolation, hence the increase was not because of an acute stress caused by the isolation procedure as such (Figure 5C). In mechanically dissected fibers, basal [Ca^2+^]_mit_ was only marginally increased after the repeated tetani irrespective of whether the fibers contracted isometrically or were allowed to shorten freely, which mimics the conditions for enzymatically dissociated fibers. However, a marked increase in basal [Ca^2+^]_mit_ after the repeated contractions were observed in fibers that were first mechanically dissected and subsequently treated with collagenase, implying that the observed effects critically depend on the collagen-containing microenvironment of the isolated fibers. Of importance, the differences in mitochondrial Ca^2+^ accumulation between mechanically dissected and enzymatically dissociated fibers were not because of differences in tetanic [Ca^2+^]_cyt_ (Figure 5D), which indicates that defective mitochondrial Ca^2+^ control rather than general alterations in cellular Ca^2+^ handling underlie the aberrant elevation in basal [Ca^2+^]_mit_ during repeated contractions in enzymatically dissociated fibers.

### The increase in basal [Ca^2+^]_mit_ in enzymatically dissociated fibers is mediated via MCU- and Ppif-dependent pathways

**Figure 6:**
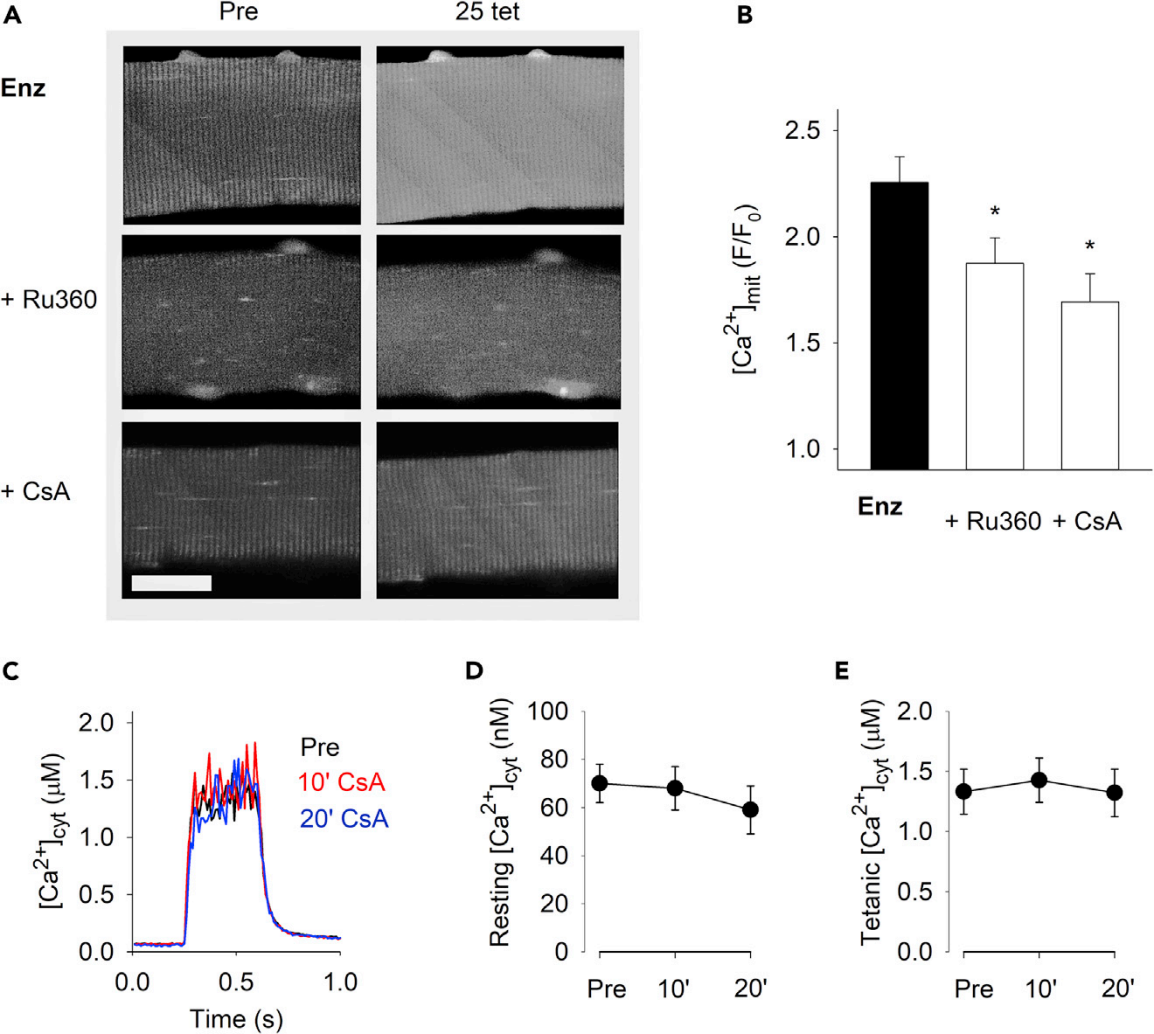
Aberrant increase in basal [Ca^2+^]_mit_ in enzymatically dissociated muscle fibers is mitigated by pharmacological inhibition of MCU and Ppif (A) Representative rhod-2 fluorescence images of enzymatically dissociated FDB fibers obtained before (Pre) and ∼5 s after 25 brief tetanic contractions produced either under control conditions or in the presence of Ru360 (microinjected plus 10 μM superfusion) or cyclosporin A (CsA; 1.6 μM). Scale bar, 20 μm. (B) Mean data of the increase in rhod-2 fluorescence after (F) relative to before (F_0_) 25 repeated tetani; ∗p < 0.05 versus control (Enz) with one-way ANOVA(n ≥ 13). (C) Superimposed average [Ca^2+^]_cyt_ records obtained in eight FDB fibers during tetanic stimulation (70 Hz, 350 ms duration) before (Pre) and after 10 and 20 min exposure to CsA (1.6 μM). Mean data of [Ca^2+^]_cyt_ at rest (D) and during the tetanic stimulation (E); one-way repeated measures ANOVA shows no effect of CsA exposure on either resting (p = 0.3) or tetanic (p = 0.5) [Ca^2+^]_cyt_ (n = 8). Experiments were performed 4 h after fiber isolation. Data are presented as mean ± SEM.

To investigate the molecular mechanisms underlying the aberrant increase in basal [Ca^2+^]_mit_ in enzymatically dissociated cells, we used the ruthenium red subcomponent Ru360 to inhibit MCU-mediated mitochondrial Ca^2+^ entry.^50^ Although Ru360 specifically inhibits MCU in experiments on isolated mitochondria, its limited plasma membrane permeability limits its use in intact cell systems.^51^ Therefore, enzymatically dissociated FDB fibers were first microinjected with Ru360 and subsequently superfused with Ru360 for 30 min before commencing the repeated tetanic stimulation. Notably, Ru360 treatment significantly decreased, but did not abrogate, the tetanic stimulation-induced increase in [Ca^2+^]_mit_ (Figures 6A and 6B).

Of interest, *Ppif* was upregulated in enzymatically dissociate fibers (see Figure 4G), and we have previously shown marked increases of PPIF/Ppif in mitochondrial myopathy patients and mice.^43^ The cyclic endecapeptide calcineurin inhibitor cyclosporin A (CsA) binds to PPIF/Ppif (cyclophilin D)^52^^,^^53^ and counteracts mPTP opening.^34^^,^^35^^,^^54^^,^^55^^,^^56^^,^^57^ CsA has previously been shown to attenuate the increase in [Ca^2+^]_mit_ in mouse mitochondrial myopathy muscle fibers exposed to repeated tetanic stimulation as well as in ischemic rabbit cardiomyocytes.^42^^,^^43^^,^^58^ In enzymatically dissociated fibers exposed to 25 repeated tetani, CsA significantly blunted the increase in basal [Ca^2+^]_mit_ (Figures 6A and 6B), and the magnitude of reduction was similar to that observed with Ru360. Measurements of [Ca^2+^]_cyt_ showed no effect of CsA either at rest or during tetanic stimulation (Figures 6C–6E).

**Figure 7:**
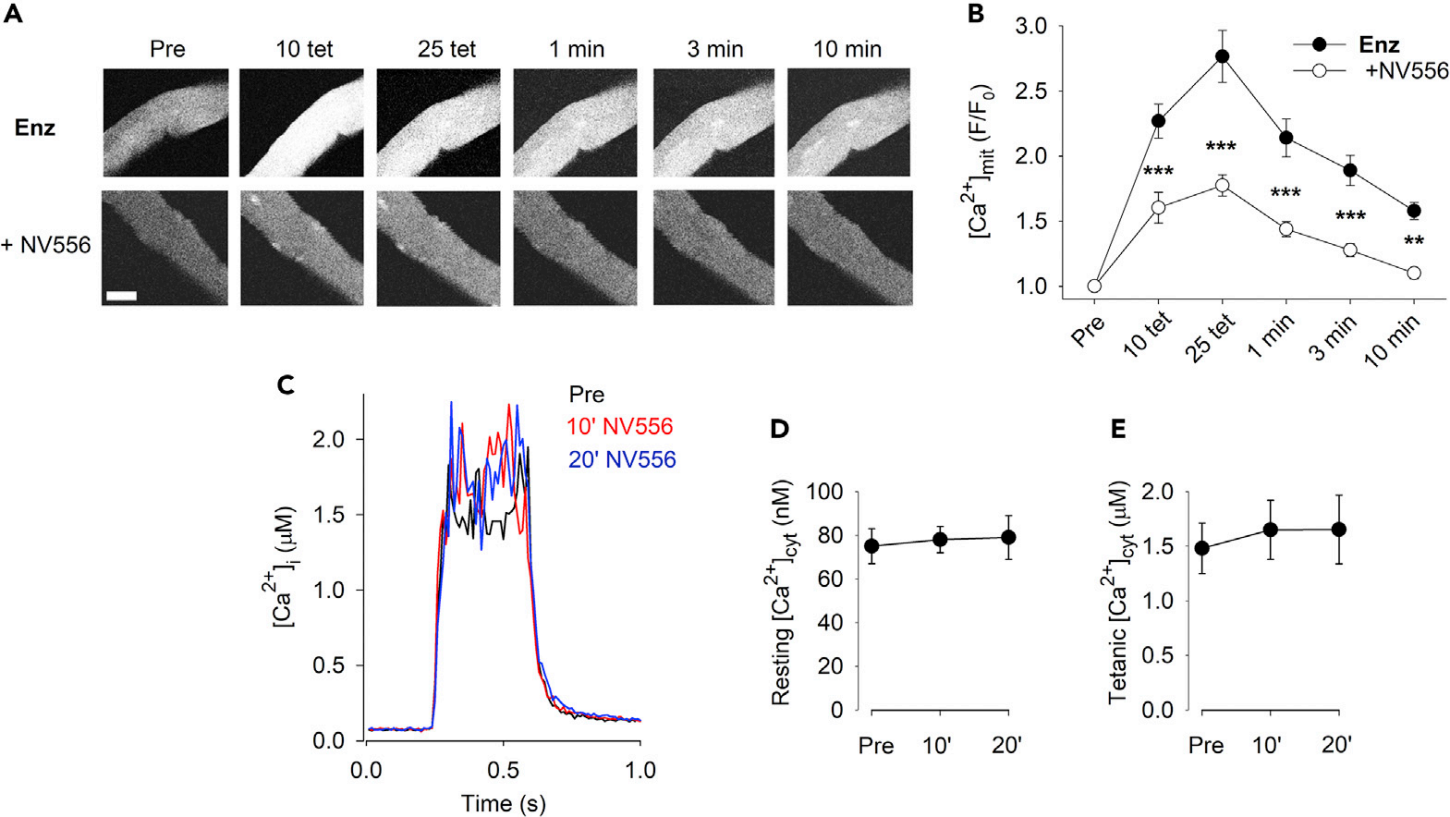
Aberrant increase in basal [Ca^2+^]_mit_ in enzymatically dissociated muscle fibers is mitigated by the cyclophilin inhibitor NV556 Representative rhod-2 fluorescence images (A; scale bar, 20 μm) and quantification of the relative increase in fluorescence (B) of enzymatically dissociated FDB fibers stimulated with 25 repeated tetani either under control conditions or in the presence of the cyclophilin inhibitor NV556 (5 μM); ∗∗p < 0.01, ∗∗∗p < 0.001 versus control with two-way repeated measures ANOVA(n ≥ 24). (C) Superimposed average [Ca^2+^]_cyt_ records obtained in nine FDB fibers during tetanic stimulation (70 Hz, 350 ms duration) before (Pre) and after 10 and 20 min exposure to NV556 (5 μM). Mean data of [Ca^2+^]_cyt_ at rest (D) and during the tetanic stimulation (E); one-way repeated measures ANOVA shows no effect of CsA exposure on either resting (p = 0.7) or tetanic (p = 0.5) [Ca^2+^]_cyt_ (n = 9). Experiments were performed 4 h after fiber isolation. Data are presented as mean ± SEM.

The above-described data suggest an important role of Ppif in mitochondrial Ca^2+^ control. To further investigate this possibility, we used the novel, specific cyclophilin inhibitor, NV556.^59^ Importantly and in agreement with the CsA results, NV556 significantly lowered basal [Ca^2+^]_mit_ during repeated tetani as well as in the subsequent recovery period (Figures 7A and 7B) without affecting [Ca^2+^]_cyt_ at rest or during tetanic stimulation (Figures 7C–7E). Thus, these results support a model in which the isolation of muscle fibers from their native microenvironment causes dysregulation of cellular organization and a partly Ppif-dependent Ca^2+^ accumulation in the mitochondrial matrix.

**Figure 8:**
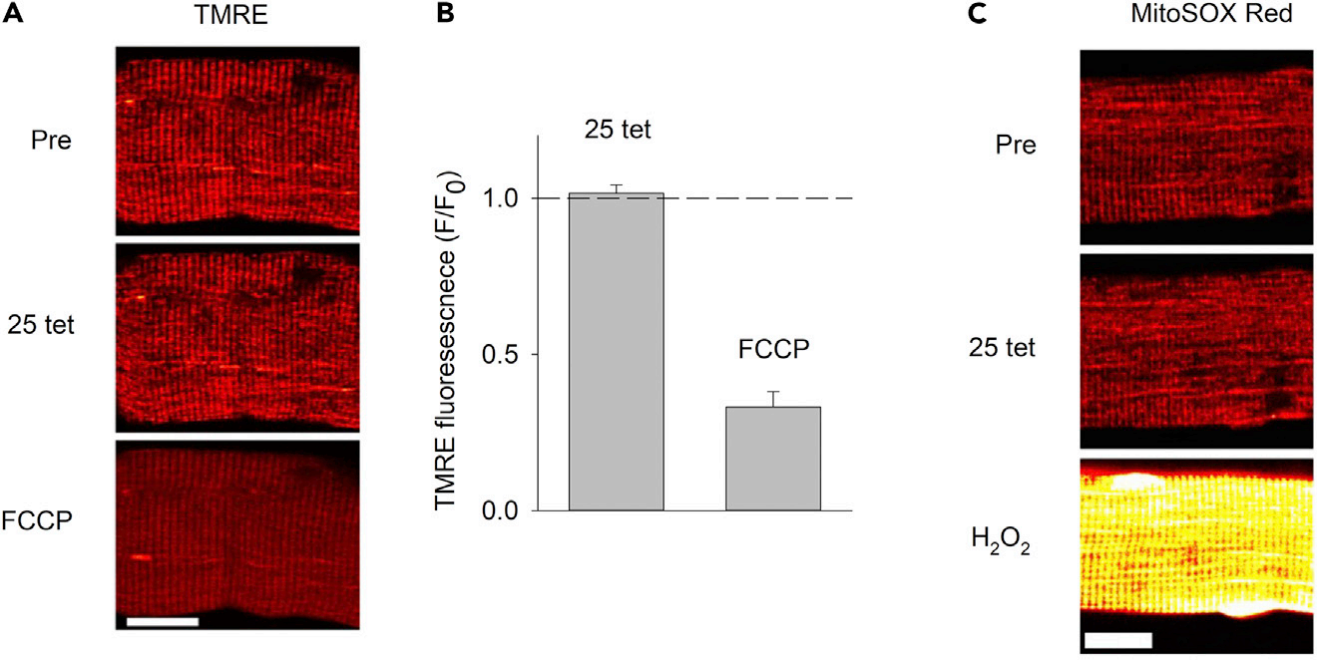
Enzymatic dissociation does not cause mitochondrial depolarization or increased ROS production (A) Representative TMRE fluorescence images and (B) mean data (±SEM) show no significant change in TMRE fluorescence ∼5 s after (F) relative to before (F_0_) 25 repeated tetani, whereas the subsequent depolarization induced by exposure to the mitochondrial uncoupler FCCP (1 μM) resulted in a marked decrease in fluorescence (n = 17). (C) Representative images show no clear increase in MitoSOX Red fluorescence ∼5 s after 25 contractions, whereas fluorescence increased several-fold during the subsequent exposure to 1 mM H_2_O_2_. Experiments were performed 4 h after fiber isolation. Scale bars in (A and C), 20 μm.

Notably and consistent with previous reports,^36^ enzymatically dissociated fibers exposed to 25 repeated tetanic contractions did not display any marked mitochondrial depolarization (Δ*ψ*_m_; measured with the fluorescent indicator TMRE) or any increase in mitochondrial reactive oxygen species (ROS) levels (measured with the fluorescent indicator MitoSOX Red) (Figure 8). These findings imply that the observed contraction-mediated increases in basal [Ca^2+^]_mit_ on enzymatic dissociation did not reach levels high enough to acutely trigger severe mitochondrial dysfunction or opening of mPTP in the high-conductance mode.

### Enzymatic dissociation masks mitochondrial Ca^2+^ handling defects in mitochondrial myopathy muscle fibers

**Figure 9:**
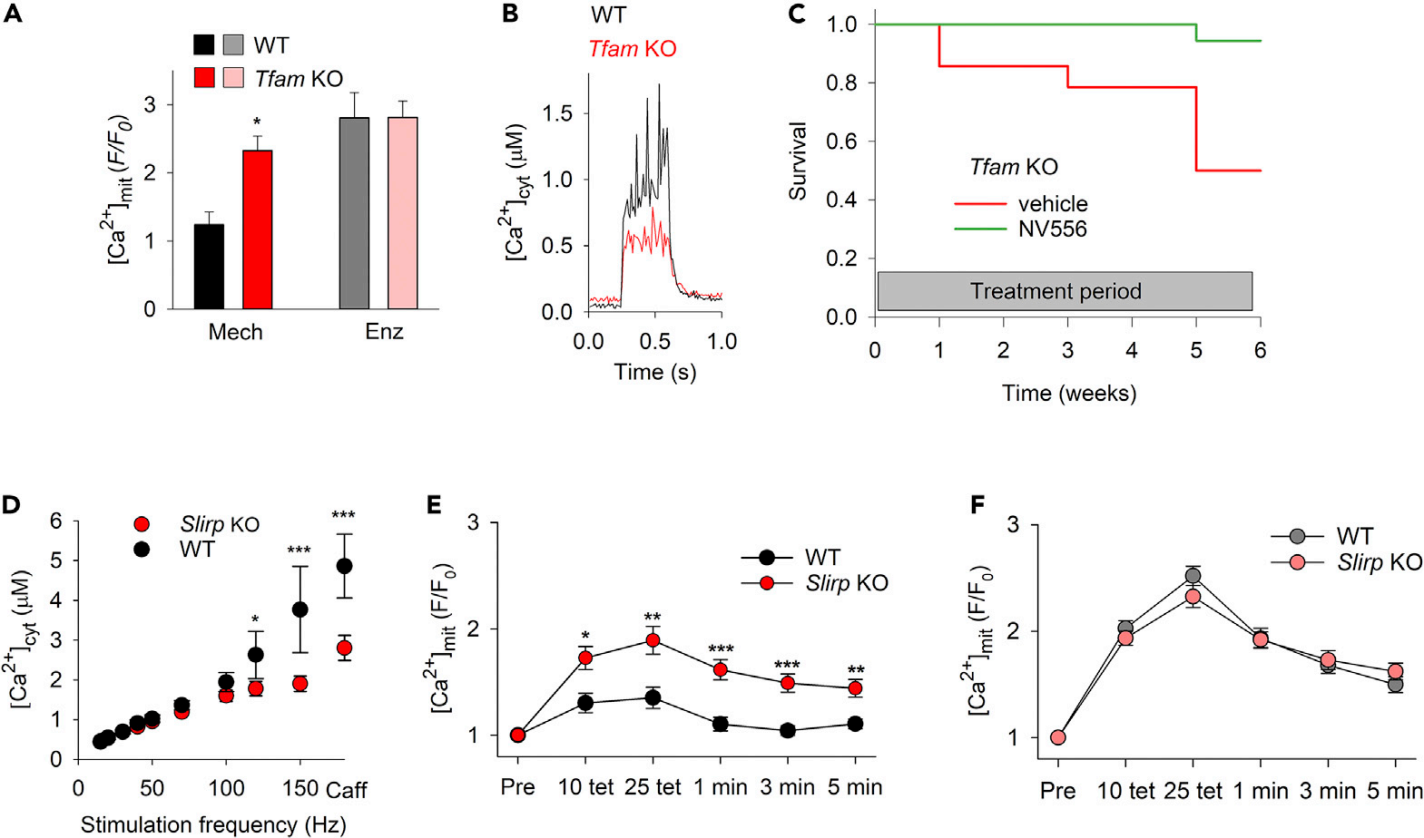
The aberrant contraction-induced increase in basal [Ca^2+^]_mit_ in mitochondrial myopathy muscle fibers eludes detection in enzymatically dissociated fibers (A) Mean data of the relative increase in rhod-2 fluorescence ∼5 s after 25 repeated tetanic contractions in mechanically dissected (Mech) Tfam KO (n = 5) and littermate controls (WT; n = 3) or enzymatically dissociated (Enz) Tfam KO (n = 10) and WT (n = 4) FDB fibers. ∗p < 0.05 versus WT with unpaired t-test. (B) Superimposed average [Ca^2+^]_cyt_ records during tetanic stimulation (70 Hz, 350 ms duration) obtained in mechanically dissected FDB fibers of control mice (WT, n = 6 fibers; same data as in Figure 5D) and Tfam KO mice (n = 4 fibers). (C) Survival curves Tfam KO mice treated with the Ppif inhibitor NV556 (140 μg daily; green line) or with vehicle control (red line) using osmotic pumps for up to 6 weeks (treatments started at an age of 14 weeks). (D) Mean data of cytosolic free [Ca^2+^] ([Ca^2+^]_cyt_) during brief stimulations at different frequencies and in the presence of caffeine (Caff, 5 mM) of WT (n = 7) and Slirp KO (n = 11) FDB fibers. Mean data of the stimulation-induced relative increase in rhod-2 fluorescence in mechanically dissected (E) and enzymatically dissociated (F) FDB fibers of WT and Slirp KO mice obtained before (Pre) and ∼5 s after 10 and 25 repeated tetanic stimulation and at 1, 3, and 5 min of recovery (n ≥ 19). Experiments were performed 4 h after fiber isolation. ∗p < 0.05, ∗∗p < 0.01, ∗∗∗p < 0.001 versus WT with two-way repeated measures ANOVA. Data are presented as mean ± SEM.

In the second part of the study, we assessed whether a contraction-induced aberrant increase in basal [Ca^2+^]_mit_ in mitochondrial myopathy muscle fibers will escape detection in enzymatically dissociated fibers. First, we used fibers from *Tfam* KO mice that display important hallmarks of severe mitochondrial myopathy.^60^ In agreement with previous results,^42^^,^^43^ repeated tetanic stimulation resulted in a marked increase in [Ca^2+^]_mit_ in mechanically dissected *Tfam* KO FDB fibers, whereas basal [Ca^2+^]_mit_ was maintained at a low level in muscle fibers of non-KO littermate controls (Figure 9A). This aberrant increase in basal [Ca^2+^]_mit_ in *Tfam* KO fibers occurred despite a reduced driving force for Ca^2+^ into the mitochondrial matrix because of lower tetanic [Ca^2+^]_cyt_ in *Tfam* KO than in control fibers (Figure 9B). Importantly, when enzymatic dissociated fibers were used, also the control non-KO fibers showed extensive elevations of basal [Ca^2+^]_mit_ after the repeated contractions and the difference in [Ca^2+^]_mit_ between *Tfam* KO and control fibers was lost. These findings corroborate the disturbed phenotype of enzymatically dissociated fibers and demonstrate that only mechanically dissected fibers can fully reveal disease-specific defects in mitochondrial Ca^2+^ handing.

To test whether Ppif inhibition would ameliorate myopathic effects, we evaluated survival of *Tfam* KO mice treated with NV556. To this end, NV556 was delivered via osmotic pumps and treatment started at an age of 14 weeks when the *Tfam* KO mice are about to enter terminal disease with severe weight loss and muscle weakness.^43^ The osmotic pumps delivered ∼140 μg NV556 per day for up to 6 weeks. At the end of the treatment period, 17 out of 18 NV556-treated mice were still alive, whereas only 7 out of 14 untreated mice were alive (p = 0.015; z-test; Figure 9C). These data agree with previous results obtained with CsA treatment^43^ and further support the critical role of Ppif in mitochondrial Ca^2+^ control. As such, they show that pharmacological inhibition of mitochondrial Ca^2+^ accumulation improves outcomes in a mouse model of lethal mitochondrial myopathy.

Intrigued by the fact that the aberrant increase in basal [Ca^2+^]_mit_ in *Tfam* KO muscle fibers eluded detection in enzymatically dissociated fibers, we performed experiments on another mouse model with defective mitochondria; that is, mice deficient of stem-loop interacting RNA binding protein (SLIRP).^61^ Despite a 50–70% reduction in the steady-state levels of mtDNA-encoded mRNAs, *Slirp* KO mice appear largely healthy with only minor (∼5%) reduction in body weight.^61^ The [Ca^2+^]_cyt_-frequency relationship was studied in FDB fibers mechanically dissected from *Slirp* KO mice and wildtype littermates by producing brief contractions at 1 min intervals. *Slirp* KO fibers displayed lower [Ca^2+^]_cyt_ than wildtype fibers at high stimulation frequencies (120–150 Hz) and during tetanic stimulation in the presence of caffeine (5 mM), which facilitates SR Ca^2+^ release and hence provides an estimate of the total amount of Ca^2+^ stored in the SR (Figure 9D).^62^ These results indicate a reduced SR Ca^2+^ storage capacity in *Slirp* KO muscle, which agrees with previous results obtained in *Tfam* KO fibers.^42^^,^^43^ Thus, a decreased SR Ca^2+^ storage capacity, which has been attributed to a decreased concentration of the SR Ca^2+^ buffering protein calsequestrin 1,^42^ is a common feature in muscle fibers of mice with two completely different genetically engineered mitochondrial defects.

In accordance with the results from *Tfam* KO muscle fibers, rhod-2 fluorescence increased more during 25 repeated tetanic contractions in mechanically dissected *Slirp* KO than in wildtype muscle fibers (Figures 9E and S2A). Thus, two mitochondrial myopathy mouse models, *Slirp* KO and *Tfam* KO, both showed aberrant basal [Ca^2+^]_mit_ elevations after repeated contractions despite decreased SR Ca^2+^ storage and hence, if anything, decreased [Ca^2+^]_cyt_-mediated driving force for mitochondrial Ca^2+^ entry during contractions. Importantly, the difference in basal [Ca^2+^]_mit_ between *Slirp* KO and wildtype fibers was masked by substantial increases in basal [Ca^2+^]_mit_ also in the wildtype group when repeated contractions were produced in enzymatically dissociated fibers (Figures 9F and S2B). Thus, isolation of wildtype muscle fibers by enzymatic ECM digestion perturbed mitochondrial Ca^2+^ handling such that the aberrant contraction-mediated mitochondrial Ca^2+^ accumulation in myopathy fibers would escape detection.

## Discussion

*In vitro* cell studies constitute essential tools for phenotype characterization, as well as for drug development. However, an increasing body of evidence highlights the fact that isolating cells by enzymatic dissociation and subsequently studying them for several days entails the rapid loss of adult cellular phenotypes, which confounds result interpretation and impairs the translation of findings.^63^ Enzymatic dissociation of cells disturbs integrin-mediated cell adhesion, which provide dynamic connections between the ECM and the intracellular cytoskeleton that are essential for the control of cell structure, including the morphology and function of mitochondria.^19^^,^^20^^,^^21^ In this study, we provide a link between enzymatic disruption of the ECM, altered cell structure, and defective control of basal [Ca^2+^]_mit_ by comparing enzymatically dissociated muscle fibers with muscle fibers isolated by mechanical dissection, which leaves the immediate ECM intact.

Enzymatic dissociation resulted in a loss of structural integrity and drastically reduced expression of genes encoding for structural proteins, such as collagens and matrix proteoglycans. Moreover, FIB-SEM revealed subtle differences in the mitochondrial 3D network with a higher number of mitochondria with individually lower volumes in enzymatically dissociated fibers, hence supporting an important role of the ECM in orchestrating the dynamic balance between mitochondrial fusion and fission.^64^^,^^65^^,^^66^ In contrast, genes associated with cell cycle and DNA replication were significantly induced in enzymatically dissociated compared to mechanically dissected fibers. These fundamental alterations were paralleled by a downregulation of markers of skeletal muscle maturation, such as genes for myosin heavy chains, in enzymatically dissociated fibers.

Although the exact link between ECM and mitochondrial phenotypes remains elusive, our results pinpoint interesting candidates for further investigations. Gene expression of the actin regulator gelsolin was maintained in mechanically dissected fibers, whereas it was among the most downregulated genes in enzymatically dissociated fibers. Gelsolin is inactive in the absence of Ca^2+^; however, on Ca^2+^ binding, gelsolin undergoes conformational changes, and the resulting activated domains participate in the severing and capping of actin filaments.^67^^,^^68^^,^^69^ Moreover, gelsolin has been shown to inhibit the cellular stress-induced increase in mitochondrial membrane permeability and loss of mitochondrial membrane potential in a Ca^2+^- and CsA-dependent manner,^70^ i.e. acting on processes that involve Ppif.^34^^,^^35^^,^^54^^,^^55^^,^^56^^,^^57^ Another tentative candidate is plectin, for which gene expression was downregulated at 4 h in enzymatically dissociated fibers. Plectin is an intermediate filament-associated protein that acts as a cytoskeletal scaffold connecting myofibrils, mitochondria and junctional complexes at the plasma membrane.^71^ We did not detect any major differences in plectin (or desmin) immunofluorescence staining between mechanically dissected and enzymatically dissociated fibers (see Figures 1E and 1F), which indicates that the cytoskeleton remained overall organized. However, subtle structural changes would not have been detected in these experiments and genetic mutations in the *PLEC* gene are associated with muscular dystrophy, characterized by the detachment of mitochondria from sarcoplasmic reticulum and mitochondrial clustering.^72^^,^^73^ Moreover, assessment of the myofibrillar structure with SHG imaging and phalloidin labeling showed a loss of distinct longitudinal ridges and valleys in enzymatically dissociated fibers, and these fibers also had longer sarcomeres than mechanically dissected fibers (see Figures 1A–1D). These results indicate that on isolation from their native microenvironment, the internal muscle fiber tension is reduced in enzymatically dissociated fibers, which might cause a rapid dedifferentiation of the adult muscle fiber phenotype. Our findings thus corroborate previous studies showing that ECM has a central role in the skeletal muscle differentiation process,^74^ and resemble observations in other tissues, such as liver, brain, and heart.^75^^,^^76^^,^^77^^,^^78^ Combined, these results argue for the importance of maintaining the physiological niche in *ex vivo* experiments of cellular function.

Rudolf et al. showed a transient increase in [Ca^2+^]_mit_ in response to the increase in [Ca^2+^]_cyt_ elicited by *in vivo* nerve stimulation of mouse tibialis anterior muscles.^8^ They showed that after a series of twitches, basal [Ca^2+^]_mit_ returned to the basal level within 100 ms. During repeated tetanic contractions, [Ca^2+^]_mit_ declined rapidly after each contraction but in a majority of fibers, it did not have time to fully return to the basal level between contractions. We here used a stimulation protocol with 350 ms tetani evoked at 2 s interval, i.e. a slightly less demanding protocol than that used by Rudolf et al. (500 ms at ∼2 s interval), and we measured basal [Ca^2+^]_mit_ ∼5 s after contractions. Thus, based on the results of Rudolf et al., only a minor increase in basal [--Ca^2+^]_mit_ after the current repeated contractions would be expected, which is in accordance with the results obtained in mechanically dissected fibers. On the other hand, the marked increase in basal [Ca^2+^]_mit_ observed in enzymatically dissociated fibers indicates a defective control of mitochondrial Ca^2+^ fluxes, i.e. a defect also observed in mechanically dissected muscle fibers of mitochondrial myopathy mouse models (see Figure 9). Notably, in the *in vivo* study of Rudolf et al.,^8^[Ca^2+^]_mit_ returned to the basal level within 10 s even after a long (2.5 s) tetanic contraction (their Figure 5K), which is in accordance with the return of basal [Ca^2+^]_mit_ to the pre-contraction level within 1 min after 25 tetani in mechanically dissected WT fibers (see Figure 9E), whereas basal [Ca^2+^]_mit_ remained markedly elevated 5 min after 25 tetani in enzymatically dissociated WT fibers (see Figures 7B and 9F).

Our results imply that the aberrant, prolonged elevation of basal [Ca^2+^]_mit_ in enzymatically dissociated fibers is Ppif-dependent as the excessive increase in basal [Ca^2+^]_mit_ after the repeated tetanic contractions was significantly decreased by the cyclophilin inhibitors CsA and NV556. In general terms, basal [Ca^2+^]_mit_ depends on the balance between mitochondrial Ca^2+^ entry and extrusion, and the Ca^2+^ buffering capacity. Ppif is an integral part of the elaborate mPTP protein complex,^34^ which may open in different conductance modes: a high-conductance state that causes collapse of the mitochondrial membrane potential, extrusion of Ca^2+^ and peptides that trigger apoptosis and ultimately cell death; a low-conductance state not accompanied by severe mitochondrial depolarization that allows additional Ca^2+^ to enter the mitochondrial matrix when [Ca^2+^]_cyt_ is increased.^58^ We did not detect any mitochondrial depolarization in enzymatically dissociated fibers during repeated contractions (see Figure 8A), which means that the driving force for Ca^2+^ was in the direction from the cytosol to the mitochondrial matrix; thus, the increase in basal [Ca^2+^]_mit_ might involve Ppif-dependent opening of mPTP in the low conductance mode. Moreover, CsA has been reported to increase mitochondrial Ca^2+^ buffering,^79^ and together with opening of the mPTP in its low-conductance mode, this provides a tentative mechanism underlying the lessened contraction induced increase in [Ca^2+^]_mit_ in enzymatically dissociated fibers. On the other hand, we are not aware of any results indicating Ppif-depent effects on mitochondrial Ca^2+^ extrusion, and our results show a slow decline of basal [Ca^2+^]_mit_ after repeated tetani both with and without Ppif inhibition (see Figure 7B).

Notably, the excessive increase in basal [Ca^2+^]_mit_ in enzymatically dissociated fibers after repeated tetanic stimulation was observed already 4 h after cell isolation, hence too soon for important changes in protein levels to develop. Thus, the excessive mitochondrial Ca^2+^ accumulation would be a direct consequence of altered mitochondrial function caused by disrupted ECM and intracellular cytoskeleton. Nevertheless, changes in gene expression 24 h after muscle fiber isolation indicate an additional enzymatic dissociation-induced long-term shift toward increased [Ca^2+^]_mit_; that is, the expression of *Mcu* and *Ppif* was higher and the expression of *Nclx* was lower in enzymatically dissociated than in mechanically dissected fibers, which would promote mitochondrial Ca^2+^ influx and limit Ca^2+^ extrusion. Of interest, previous studies showed that myopathies in mice deficient in the ECM protein collagen VI could be counteracted by CsA^32^ and the non-immunosuppressive Ppif inhibitor alisporivir (also called Debio 025).^33^ Furthermore, our finding that pharmacological inhibition of aberrant mitochondrial Ca^2+^ accumulation improved survival in the *Tfam* KO mouse model of lethal mitochondrial myopathy supports a scheme where the adverse effects of ECM perturbations are mediated, at least in part, via impaired Ppif-dependent fine-tuning of cytosolic-mitochondrial Ca^2+^ fluxes, thus providing a molecular explanation for prolonged survival previously reported when *Tfam* KO mice were treated with CsA,^43^ as well as treatment with the more specific cyclophilin inhibitor NV556 used in the present study.

The presented findings highlight multiple important implications for the study of cell biological phenomena *in vitro*. Firstly, our data emphasize the importance of using cell culture systems that preserve the immediate ECM to ensure that results faithfully reflect *in vivo* processes. Secondly, enzymatic digestion rapidly changes the molecular phenotypes and functionality of cells. For instance, recent studies have shown redundant activation of muscle stem cells isolated from adult skeletal muscle with standard enzymatic dissociation protocols, which has important consequences for the use of these cells as quiescent controls.^80^^,^^81^ Thirdly, dissolving the microphysiological niche around cells can result in perturbations that resemble pathological phenotypes observed in mitochondrial disease, providing further evidence for an intricate interplay between cellular structure, Ca^2+^ fluxes, metabolism, and function. Specifically, the pathognomonic mitochondrial Ca^2+^ accumulation during repeated contractions of muscle fibers in mitochondrial myopathy would be missed in experiments performed on enzymatically dissociated cells. Thus, enzymatically dissociated cells should be avoided as an experimental paradigm for the study of diseases that potentially involve altered mitochondrial Ca^2+^ signaling.

In conclusion, disruption of the organotypic niche results in the loss of structural integrity of muscle fibers accompanied by impaired control of mitochondrial Ca^2+^. The molecular link between the processes involves a Ppif-dependent mitochondrial Ca^2+^ accumulation resembling that observed in mitochondrial myopathies. Our results support a central role of mitochondrial Ca^2+^ as a critical mediator that connects the native extracellular microenvironment to the maintenance of normal cellular structure and function.

### Limitations of the study

We show clear morphological and functional differences between enzymatically dissociated and mechanically dissected fibers, and we attribute these to disruption of the extracellular matrix with collagenase treatment. However, there are inevitably other methodological differences between the two groups, such as, the strain imposed by tendons and connective tissue during contractions of mechanically dissected fibers, which are absent in enzymatically dissociated fibers. To deal with this, experiments were performed where mechanically dissected fibers were allowed to shorten freely during contractions (i.e., without mechanical stress via the tendons) or where mechanically dissected fibers were subsequently treated with collagenase and contractions performed under the same conditions as enzymatically dissociated fibers. Nevertheless, we cannot exclude that the observed differences between mechanically dissected and enzymatically dissociated fibers involved other methodological aspects; for instance, mechanically dissected fibers were not exposed to the trituration process.

Our results imply that contraction-induced mitochondrial Ca^2+^ accumulation in enzymatically dissociated fibers occurs partly via a Ppif-dependent pathway. Cyclophilin inhibitors act on Ppif to inhibit opening of mPTP. In situations of severe cellular stress, mPTP enters a high-conductance state. In the present study, however, the acute stress is relatively mild, and the high-conductance state is not entered. We propose that the cyclophilin inhibitors then act by inhibiting opening of a low-conductance state or by increasing the mitochondrial Ca^2+^ buffering capacity, but further experiments are required to clarify the exact details of their action.

## STAR★Methods

### Key resources table

**Table:** 

REAGENT or RESOURCE	SOURCE	IDENTIFIER
**Chemicals, peptides, and recombinant proteins**		

NV556	Abliva AB, Lund, Sweden	N/A
Collagenase type 1	Merck KGaA, Darmstadt, Germany	Cat# SCR103
Antibiotic antimycotic solution	Merck KGaA, Darmstadt, Germany	Cat# A5955

**Experimental models: Organisms/strains**		

C57BL/6JRj mice	Janvier Labs, France	N/A
Fast-twitch skeletal muscle fiber-specific *Tfam* KO mice	(Wredenberg et al., 2002)^60^	N/A
*Slirp* KO mice	(Lagouge et al., 2015)^61^	N/A

**Software and algorithms**		

MATLAB	MathWorks, Natick, MA	N/A
TEM Image & Analysis software ver. 4.17	Thermo Fisher Scientific	N/A
Auto Slice & View 4 Software	Thermo Fisher Scientific	N/A
ImageJ	https://imagej.nih.gov/ij/	N/A
IMOD software package ver. 4.9.13	https://bio3d.colorado.edu/imod/	Kremer et al., 1996^82^
Qlucore Omics Explorer	Qlucore, Lund, Sweden	N/A
WebGestalt Toolbox	http://www.webgestalt.org/	Liao et al., 2019^83^
SigmaPlot 13	Systat Software Inc, CA	N/A
RTA3.4.4 pipeline and Bcl2fastq (v2.20) conversion software	Illumina	N/A
Stringtie nf-core/rnaseq package	https://nf-co.re/rnaseq/usage	N/A
BioRender	https://biorender.com	N/A

### Resource availability

#### Lead contact

Further information and requests for resources and reagents should be directed to and will be fulfilled by lead contact, Håkan Westerblad (hakan.westerblad@ki.se).

#### Materials availability

This study did not generate new unique reagents.

### Experimental model and subject details

All animal experiments were approved by the Stockholm North Local Animal Ethics Committee and complied with the Swedish Welfare Ordinance, and applicable regulations and recommendations from Swedish authorities. Mice were housed at room temperature (∼22°C) under a 12-h light/dark cycle. Adult mice were used, except when stated otherwise. C57BL/6JRj (Janvier Labs) female mice were used, except when stated otherwise. Fast-twitch skeletal muscle fiber-specific *Tfam* KO mice and their controls were generated as described previously.^60^*Slirp* KO mice and wildtype littermates were generated as described previously.^61^ Male and female *Tfam* KO and *Slirp* KO mice were used. Mice were euthanized by rapid neck dislocation and the FDB muscles were excised.

Some 14 weeks old *Tfam* KO mice were treated with the specific cyclophilin inhibitor, NV556,^59^ or vehicle only. NV556 was dissolved to 40 mg/mL in cyclodextrin formulation (pH 7.4). Mini-osmotic pumps (Alzet model 2006) were loaded with approximately 200 μL of either dissolved NV556 or cyclodextrin formulation only and incubated in sterile phosphate-buffered saline for 48 h prior to implantation. The mini-osmotic pumps were implanted subcutaneously on the back of mice under isoflurane anesthesia. Mice were weighed 48 h after surgery; body weight and health status were then monitored twice per week for the remainder of the experiment period. Mice were regarded as terminally ill and euthanized either at loss of 20% body weight or severe loss of muscle functionality.

### Method details

#### Isolation of muscle fibers

##### Enzymatic dissociation

*Flexor digitorum brevis* (FDB) muscles were cleaned of tendons, connective tissue, and blood vessels and incubated for ∼2 h at 37°C in 0.3% collagenase type 1 (Merck) in Dulbecco's modified Eagle medium (DMEM; Invitrogen) supplemented with 20% fetal bovine serum. Muscles were then transferred to fresh DMEM and gently triturated to dissociate individual muscle fibers. A volume of 300 μL of the resultant muscle fiber suspension was placed in laminin-coated glass-bottom Petri dishes, and fibers were allowed to attach for 15 min. Thereafter, 3 mL DMEM supplemented with antibiotic antimycotic solution (1 μL/mL, Merck) was added. Experiments were performed ∼4 h after enzymatic dissociation, except when otherwise noted.

##### Mechanical dissection

Single or small bundles (2–5 fibers) of FDB fibers were mechanically dissected as previously described.^41^ Aluminum clips were attached to each of the trimmed tendons and allowing the preparation to be anchored on the bottom of a glass-bottomed petri dish by coating the clips with silicone grease and pressing them against the glass. Importantly, fibers were never stretched during or after this procedure except when they were used in experiments with isometric contractions. In one set of experiments (see Figure 5C), dissected fibers were subjected to the same enzymatic dissociation protocol as the dissociated fibers with the exception that no trituration was performed.

#### Second Harmonic Generation microscopy

Mechanically dissected and enzymatically dissociated FDB fibers were prepared as described above and fixed in 2.5% glutaraldehyde (TAAB Laboratories). They were then imaged using a multi-photon microscope (TriMScope II, LaVision BioTech, Bielefeld, Germany) equipped with a combination of two water immersion objectives (LD C-APO 40×/1.1 W Corr M27 on the excitation side, W Plan-APO 20x/1.0 DIC M27 on the transmission side, Zeiss) and a mode-locked ps-pulsed Ti:Sa-laser (Chameleon Vision II, Coherent, Santa Clara, CA) tuned to 810 nm to excite the myosin SHG signal.^46^^,^^84^ The SHG signal from myosin was separated from other (autofluorescence) signals by a band-pass filter (405/20 nm, CHROMA, Bellows Falls, VT) and detected by a non-descanned photomultiplier (H7422-40, Hamamatsu Photonics, Hamamatsu, Japan). Muscle fibers were z-scanned (voxel-size: 0.14 × 0.14 × 0.3 μm) to detect the cosine angle sum based on boundary tensor analysis.^44^^,^^45^ Image processing was performed in MATLAB (MathWorks, Natick, MA).

#### Immunofluorescence microscopy

Mechanically dissected or enzymatically dissociated muscle fibers were fixed with 4% methanol-free PFA for 2 h at 4°C. Afterward, fibers were washed 3x with PBS and permeabilized with 0.1% Triton X-100 in PBS at room temperature. After repeated PBS washes, the fibers were incubated with 1:50 rabbit anti-desmin (Abcam; ab15200) or 1:50 rabbit anti-plectin (Huabio; ET1607-80) antibodies overnight at 4°C. The primary antibodies were extensively washed out and samples were incubated with AlexFluor488-conjugated secondary goat anti-rabbit antibody for 2h at room temperature. Images were collected on a Zeiss LSM800-Airy confocal microscope.

#### Transmission electron microscopy

Mechanically dissected and enzymatically dissociated FDB muscle fibers were prepared as described above and fixed in 2.5% glutaraldehyde (TAAB Laboratories), 4% formaldehyde in 0.1 M sodium cacodylate buffer. All samples were processed using Pelco Biowave Pro + microwave tissue processor (Ted Pella) according to,^85^ with minor modifications: no Ca^2+^ was used during the fixation and contrasting steps with lead aspartate were omitted to reduce overstaining. Samples were trimmed with a glass knife and 70 nm ultrathin sections were picked up on Cu-grids and examined with the TEM Talos L120C (FEI, currently Thermo Fischer Scientific) operating at 120 kV. Micrographs were acquired with a Ceta 16M CCD camera using TEM Image & Analysis software ver. 4.17 (Thermo Fisher Scientific).

For focused ion beam scanning electron microscopy (FIB-SEM), a small cube of the sample was cut and glued on an SEM stub with epoxy and silver glue. In the SEM chamber, the specimen was coated with a 5 nm thin layer of platinum to reduce charging. Specimens were imaged using Scios DualBeam SEM and the ‘Auto slice and view 4’ software system (Thermo Fisher Scientific); the electron beam operated at 2 kV and 0.2 nA and was detected with T1 in-lens detector. A 1 μm protective layer of platinum was deposited on the selected area before milling. FIB milling thickness was set to 30 nm and each slice was imaged with pixel sizes 3.55 × 3.55 nm (for mechanically dissected fiber) and 3.74 × 3.74 nm (for enzymatically dissociated fiber). Images were further processed using the ImageJ plugins ‘Linear Stack Alignment with SIFT’ and ‘Multistackreg’ (https://imagej.nih.gov/ij/) and the mitochondrial network was reconstructed and analyzed in a final volume of 365 μm^3^ (7.9 × 4.5 × 10.3 μm). Identified mitochondrial volumes were modeled and measured using the IMOD software package ver. 4.9.13.^82^

#### RNA-sequencing

RNA sequencing by poly-A capture was performed using >10 ng RNA input material. Image analysis, base calling and quality checks were performed using the RTA3.4.4 pipeline and Bcl2fastq (v2.20) conversion software (Illumina). Quality control, removal of genomic contaminants and ribosomal RNA, UMI-based read deduplication, transcript assembly and quantification were conducted using the Stringtie nf-core/rnaseq package (https://nf-co.re/rnaseq/usage). Genes with an average number of fragments per kilo base per million mapped reads (FPKM) > 0.5 across all samples were analyzed using Qlucore Omics Explorer (Lund, Sweden). Differential gene expression analysis was conducted using DESeq2 and multiple testing correction was applied using the Benjamini–Hochberg procedure with false discovery rates (FDRs) ≤ 5%. Pathway enrichment analysis was conducted based on the PANTHER gene family classification system using the WebGestalt toolbox.^83^ Gene expression values (FPKM) of all samples are presented in Table S1.

#### Confocal measurements with fluorescent indicators

For measurements of mitochondrial [Ca^2+^] ([Ca^2+^]_mit_), membrane potential (Δ*ψ*_m_) and ROS production (see below), unmounted FDB fibers were loaded with fluorescent indicators for 20minat room temperature. During FDB fiber experiments, cells were superfused at room temperature (∼25°C) with Tyrode solution (in mM): NaCl, 121; KCl, 5.0; CaCl_2_, 1.8; MgCl_2_, 0.5; NaH_2_PO_4_, 0.4; NaHCO_3_, 24.0; EDTA, 0.1; glucose, 5.5; 0.2% fetal calf serum. The solution was bubbled with 95% O_2_–5% CO_2_. Measurements were performed using a BioRad MRC 1024 confocal unit with a dual Calypso laser (Cobolt, Solna, Sweden) attached to a Nikon Diaphot 200 inverted microscope. Confocal images were obtained before, ∼5 s after 10 and 25 repeated tetani (70 Hz, 350 ms stimulation trains given at 2 s intervals), and at regular intervals after the contractions. Note that confocal imaging was always performed at rest and not during an ongoing contraction due to fiber movement. Confocal images were analyzed using ImageJ and data are expressed as *F*/*F*_0_, i.e., the ratio of the fluorescence intensity after and before the repeated contractions, respectively.

For measurements of [Ca^2+^]_mit_, fibers were incubated in 5 μM rhod-2 in the membrane permeable AM form (Invitrogen). Rhod-2 was excited with 531 nm light and the emitted light was collected through a 585 nm long-pass filter.

For measurements of the mitochondrial membrane potential (Δ*ψ*_m_), fibers were incubated in 1 μM TMRE (Invitrogen). TMRE was excited at 531 nm and the emitted light was collected through a 605 nm long-pass filter. Approximately 30 min after completion of the 25 repeated tetani, fibers were exposed to 1 μM FCCP (Sigma-Aldrich) to significantly depolarize the mitochondria and confocal images were obtained.

For measurements of mitochondrial ROS production, fibers were loaded with 5 μM MitoSOX Red (Invitrogen). MitoSOX Red was excited with 488 nm light and emitted light was collected through a 585 nm long-pass filter. As a positive control, 1 mM H_2_O_2_ was applied at the end of experiments to increase mitochondrial superoxide by inducing product inhibition of superoxide dismutase 2,^86^^,^^87^ and confocal images were obtained every minute until the MitoSOX Red signal reached a plateau.

##### Ru360, CsA and NV556 experiments

Enzymatically dissociated FDB fibers were loaded with rhod-2 to measure [Ca^2+^]_mit_ as described above. To investigate the potential sites of Ca^2+^ entry into mitochondria, enzymatically dissociated fibers were exposed to either Ru360 (Calbiochem), CsA or NV556 (Abliva AB, Lund, Sweden). Ru360 was first injected into the fibers and they were subsequently superfused with 10 μM Ru360 throughout the experiment. CsA (1.6 μM; Novartis) or NV556 (5 μM) was applied to the fibers for 5 min before and during the 25 repeated tetani.

#### Single fiber [Ca^2+^]_cyt_ measurements

Intact single FDB fibers were mechanically dissected.^41^ Aluminum or platinum clips were attached to the tendons and the fiber was mounted in a chamber between an Akers 801 force transducer (Kronex Technologies, Oakland, CA, USA) and an adjustable holder and subsequently superfused by Tyrode solution (see above) at room temperature. The fiber length was adjusted to obtain maximum tetanic force. The fiber was stimulated with supramaximal electrical pulses (0.5 ms in duration) delivered via platinum electrodes placed along the long axis of the fiber. The steady-state [Ca^2+^]_cyt_-frequency relationship was obtained by stimulating fibers for 350 msat 15–150 Hz every 1 min; 150 Hz contractions were also produced in the presence of 5 mM caffeine to assess the SR Ca^2+^ storage capacity.

We used the relatively high-affinity fluorescent indicator indo-1 to measure [Ca^2+^]_cyt_. An advantage with high-affinity Ca^2+^ indicators is that they can measure resting as well as mean tetanic [Ca^2+^]_cyt_. However, they cannot readily detect fast transient [Ca^2+^]_cyt_ increases and hence tetanic [Ca^2+^]_cyt_ values tend to be lower with high-affinity than with low-affinity Ca^2+^ indicators. On the other hand, low-affinity Ca^2+^ indicators cannot accurately measure the low [Ca^2+^]_cyt_ at rest.^88^ Fibers were microinjected with indo-1 (Molecular Probes/Invitrogen, Carlsbad, CA, USA), or loaded with indo-1 AM in experiments on enzymatically dissociated fibers. The emitted fluorescence of indo-1 was measured with a system consisting of a Xenon lamp, a monochromator, and two photomultiplier tubes (Photon Technology International, Wedel, Germany). The excitation light was set to 360 nm, and the light emitted at 405 ± 5 and 495 ± 5 nm was measured by the photomultipliers. The ratio of the light emitted at 405 nm to that at 495 nm (R) was converted to [Ca^2+^]_cyt_ using the following equation:

$${\left[{Ca}^{2+}\right]}_{cyt}=K_{d}\times \beta \times \frac{\left({R-R}_{\min}\right)}{\left(R_{\max}-R\right)}$$

where K_d_ is the apparent dissociation constant of indo 1, β is the ratio of the 495 nm signals at very low and saturating [Ca^2+^]_cyt_, R_min_ is the ratio at very low [Ca^2+^]_cyt_, and R_max_ is the ratio at saturating [Ca^2+^]_cyt_. Fluorescence was sampled online and stored on a computer for subsequent data analysis. [Ca^2+^]_cyt_ was measured at rest and as the mean during 70 Hz, 350 ms tetanic stimulation. The steady-state [Ca^2+^]_cyt_-frequency relationship was obtained in *Slirp* KO and control WT fibers by stimulating fibers for 350 msat 15–150 Hz every 1 min; 150 Hz contractions were also produced in the presence of 5 mM caffeine to assess the SR Ca^2+^ storage capacity.

### Quantification and statistical analysis

Statistical analyses were performed with SigmaPlot 13 (Systat Software Inc, CA). Student’s paired, unpaired t-tests, one-way ANOVA, or z-test were used as appropriate. Two-way repeated measures ANOVA was used to determine differences between two groups of repeatedly stimulated fibers. The Holm-Sidak post-hoc analysis was used when ANOVA showed a significant difference between groups. Significance was assumed for p< 0.05. Data are presented as mean ± SEM.

## Data Availability

• RNA-sequencing data of all samples are presented in Table S1. • This paper does not report original code. • Any additional information required to reanalyze the data reported in this paper is available from the lead contact upon request.

## Supplemental information

Document S1. Figures S1 and S2

Table S1. Gene expression values (FPKM) of all samples related to RNAsequencing experiments presented in Figures 3 and 4
