# Supplementary material for: Enzymatically dissociated muscle fibers display rapid dedifferentiation and impaired mitochondrial calcium control
Source: iScience. 2022 Nov 22;25(12):105654. doi: 10.1016/j.isci.2022.105654 (PMC9720020; doi:10.1016/j.isci.2022.105654)
Supplement: Document S1. Figures S1 and S2 [file mmc1.pdf]

iScience, Volume *25*

Supplemental information

Enzymatically dissociated muscle fibers display rapid dedifferentiation and impaired mitochondrial calcium control

Charlotte Gineste, Sonia Youhanna, Sabine U. Vorrink, Sara Henriksson, Andrés Hernández, Arthur J. Cheng, Thomas Chaillou, Andreas Buttgereit, Dominik Schneidereit, Oliver Friedrich, Kjell Hultenby, Joseph D. Bruton, Niklas Ivarsson, Linda Sandblad, Volker M. Lauschke, and Håkan Westerblad

Figure S1

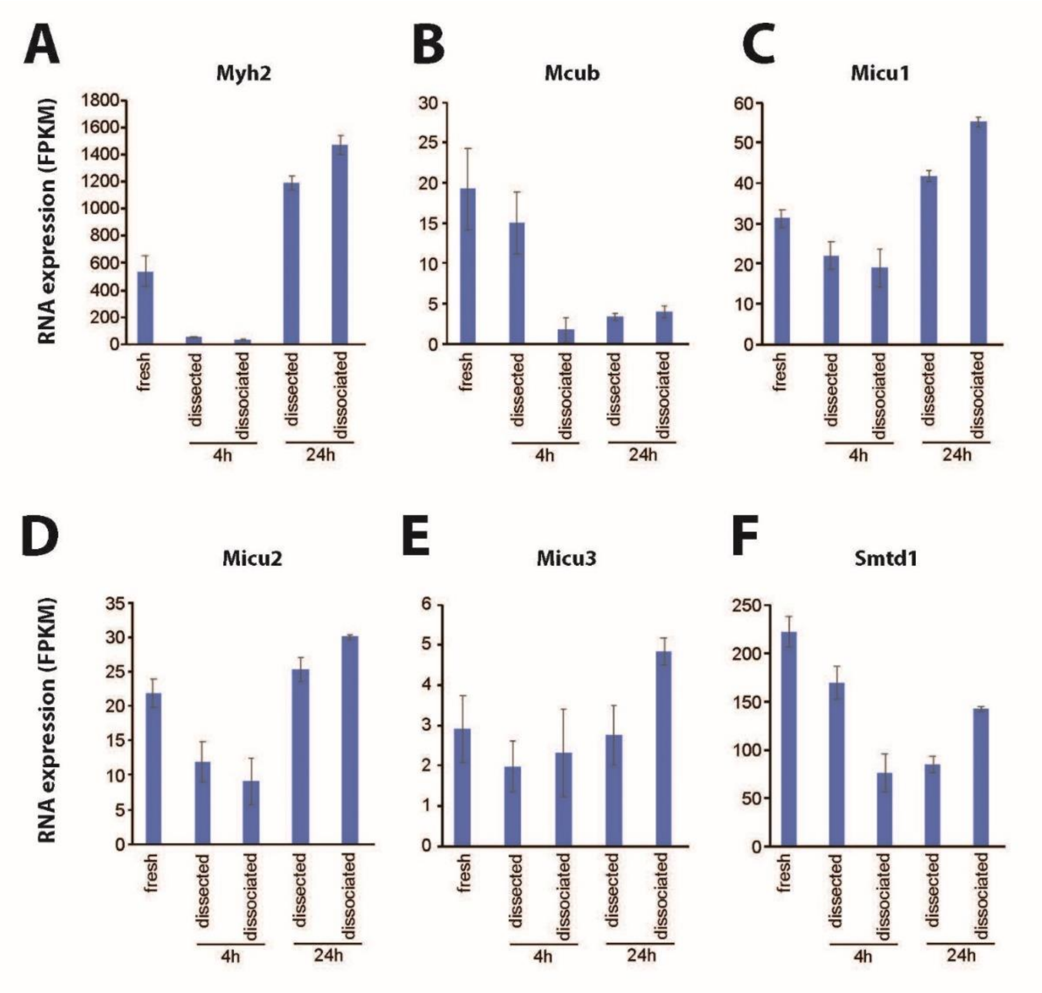

Enzymatic dissociation had no consistent effect on the mRNA expression of myosin heavy chain IIA or Mcu regulators; related to RNA-sequencing experiments and Figure 4.
Expression of genes encoding for myosin heavy chain IIA (Myh2; **A**) and Mcu regulators: mitochondrial Ca^{2+} uniporter B (Mcub; **B**); mitochondrial Ca^{2+} uptake proteins 1-3 (Micu1-3; **C-E**); and essential Mcu regulator (EMRE, Smtd1; **F**). Data are presented as mean ± SEM; n=4.

Figure S2

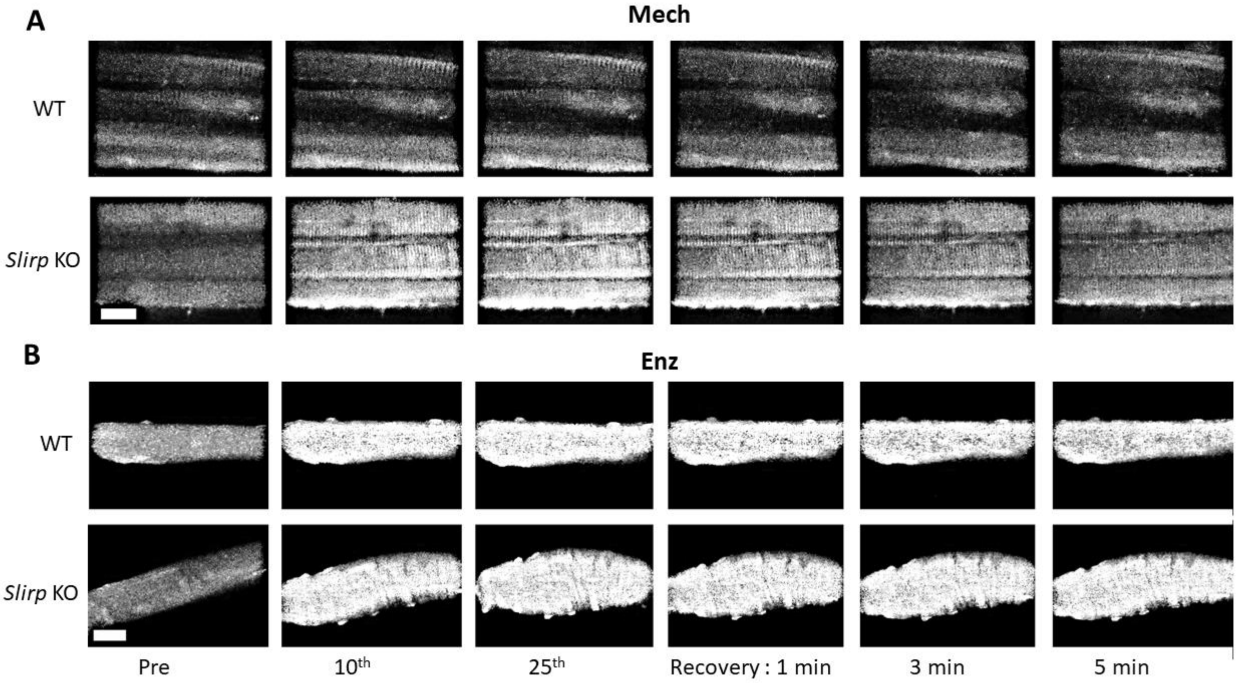

The aberrant contraction-induced increase in $[Ca^{2+}]_{mit}$ in mitochondrial myopathy muscle fibers eludes detection in enzymatically dissociated fibers; related to Figure 9, panels E and F. Representative rhod-2 fluorescence images of a small bundle of mechanically dissected fibers (**A**) and enzymatically dissociated (**B**) FDB fibers of WT and *Slirp* KO mice obtained before (Pre), after 10 and 25 repeated tetanic stimulations, and at 1, 3, and 5 minutes of recovery (scale bars, 20 µm). Experiments were performed 4 hours after fiber isolation.
